# Genetic polymorphism of the iron-regulatory protein-1 and -2 genes in age-related macular degeneration

**DOI:** 10.1007/s11033-012-1539-6

**Published:** 2012-02-14

**Authors:** Ewelina Synowiec, Magdalena Pogorzelska, Janusz Blasiak, Jerzy Szaflik, Jacek Pawel Szaflik

**Affiliations:** 1 Department of Molecular Genetics, University of Lodz, Pomorska 141/143, 90-236 Lodz, Poland; 2 Department of Ophthalmology, Medical University of Warsaw and Samodzielny Publiczny Kliniczny Szpital Okulistyczny, Sierakowskiego 13, 03-710 Warsaw, Poland

**Keywords:** Age-related macular degeneration, AMD, IRP-1 and -2, Gene polymorphism, Iron, Oxidative stress, Reactive oxygen species, Iron-regulatory proteins

## Abstract

Iron can be involved in the pathogenesis of AMD through the oxidative stress because it may catalyze the Haber–Weiss and Fenton reactions converting hydrogen peroxide to free radicals, which can induce cellular damage. We hypothesized that genetic polymorphism in genes related to iron metabolism may predispose individuals to the development of AMD and therefore we checked for an association between the g.32373708 G>A polymorphism (rs867469) of the *IRP1* gene and the g.49520870 G>A (rs17483548) polymorphism of the *IRP2* gene and AMD risk as well as the modulation of this association by some environmental and life-style factors. Genotypes were determined in DNA from blood of 269 AMD patients and 116 controls by the allele-specific oligonucleotide-restriction fragment length polymorphism and the polymerase chain reaction-restriction fragment length polymorphism. An association between AMD, dry and wet forms of AMD and the G/G genotype of the g.32373708 G>A-*IRP1* polymorphism was found (OR 3.40, 4.15, and 2.75). On the other hand, the G/A genotype reduced the risk of AMD as well as its dry or wet form (OR 0.23, 0.21, 0.26). Moreover, the G allele of the g.49520870 G>A-*IRP2* polymorphism increased the risk of the dry form of the disease (OR 1.51) and the A/A genotype and the A allele decreased such risk (OR 0.43 and 0.66). Our data suggest that the g.32373708 G>A-*IRP1* and g.49520870 G>A-*IRP2* polymorphisms may be associated with increased risk for AMD.

## Introduction

Age-related macular degeneration (AMD) is an eye disease that is characterized by progressive vision loss of variable severity accompanied by distinct changes in the structure of the retina, retinal pigment epithelium (RPE), Bruch’s membrane and choriocapillaris [1]. Currently, AMD is estimated to affect approximately 30 million people globally and this number is expected to triple over the next 25 years [2]. AMD ranks third among the global causes of visual impairment with a blindness prevalence of 8.7%. It is the primary cause of visual deficiency in industrialized countries [3].

Although the pathophysiology of AMD is not clearly understood, it is well accepted that both oxidative stress and genetic factors in combination with environmental and life-style factors play an important role in the pathogenesis of the disease. The most consistent major risk factor for AMD is age. The development of AMD is a slow progressive process that occurs with aging and mainly affects people over age 60 [4]. While people in middle age have about a 2% risk of getting AMD, the risk increased to nearly 30% in those over age 75 [5]. Also, many other risk factors, including smoking, female sex, obesity, atherosclerosis, white race, exposure to sunlight and consumption of a non-balanced diet, have been proposed for AMD development [6, 7]. Results of several studies have shown that AMD affects Caucasians more than other populations [8–10].

Age-related disorders, including retinal diseases may result from cumulative oxidative damage resulting from reactive oxygen species (ROS) [11–13]. The macula is a source of high metabolic activity and is therefore exposed to high levels of ROS. With age, the balance between the production of ROS and local antioxidant levels can be disturbed, resulting in oxidative damage to outer segments of photoreceptors and lead to a progressive deterioration of the RPE [14]. One source of ROS generation is iron. Iron may catalyze the Haber–Weiss and Fenton reactions converting hydrogen peroxide to free radicals, which can cause oxidative tissue damage. AMD may be exacerbated by retinal iron overload and histopathology of eyes with macular degeneration has shown elevated levels of iron in RPE, Bruch’s membrane and within drusen [15, 16]. Moreover, the concentration of retinal iron increases with age [17]. Iron homeostasis is controlled by the regulation of the expression of iron-regulatory proteins (IRPs), which can bind iron-responsive elements (IREs) on the mRNA of target proteins.

A number of epidemiological studies have implicated AMD as an inherited disease showing that family members are at an increased risk of the disease [18–20]. Considerable evidence in family, twin and sibling studies exists, suggesting a genetic basis of AMD. Twin studies have demonstrated that genetic factors contribute to 46–71% of the overall variation in severity of macular degeneration. When one or both twins have AMD, concordance for AMD is 55% among monozygotic twins and 25% among dizygotic twins [21]. Several family studies have shown that patients with a positive family history of AMD are at increased risk of this disease [22–24]. Interestingly, Luo et al. [25] identified 4,764 patients with AMD and examined familial aggregation and risk of AMD in a Utah population using a population-based case–control study. The results showed that the population-attributable risk for AMD was calculated to be 0.34. Recurrence risks in relatives indicate increased relative risk in siblings (2.95), first cousins (1.29), second cousins (1.13), and parents (5.66) of affected individuals.

In recent years, the role of genetic factors in AMD development has been extensively investigated. Numerous case–control studies have confirmed the association between single nucleotide polymorphisms (SNPs) and AMD [26–28]. We hypothesized that genetic polymorphisms in the IRPs genes may be associated with development of AMD. To test our hypothesis, we evaluated the association between the g.32373708 G>A polymorphism (rs867469) of the *IRP1* gene and the g.49520870 G>A (rs17483548) polymorphism of the *IRP2* gene and AMD as well as the modulation of this association by some environmental and life-style factors. Both polymorphisms are SNPs with a minor allele frequency >3% in European population and are located in the 5′ flanking region of these genes. Polymorphism in this region can affect mRNA stability and degradation, and gene expression. [29].

## Materials and methods

### Study subjects and data collection

This case–control study included a total of 270 patients with AMD and 116 disease-free control subjects. Among AMD patients, 100 had dry AMD and the remaining 170—wet form of this disease. Eight patients with the dry form of the disease had geographic atrophy. The control subjects had no clinical evidence of AMD after undergoing the same comprehensive ophthalmic examination that was performed to confirm AMD in the patient group. Medical history was obtained from all subjects and no one reported current or previous cancer or any genetic disease. All patients and controls were examined in the Department of Ophthalmology, Medical University of Warsaw (Warsaw, Poland). They underwent ophthalmic examination, including best-corrected visual acuity, intraocular pressure, slit lamp examination, and fundus examination, performed with a slit lamp equipped with either non-contact or contact fundus lenses. Diagnosis of AMD was confirmed by optical coherence tomography (OCT) and, in some cases, by fluorescein angiography (FA) and indocyanin green angiography (ICG). OCT evaluated retinal thickness, the presence of RPE atrophy, drusen, or subretinal fluid and intraretinal edema; angiography assessed the anatomical status of the retinal vessels, the presence of choroidal neovascularization and leakage. The OCT examinations were performed with Stratus OCT model 3000, software version 4.0 (Oberkochen, Germany). The FA and ICG examinations were performed with a Topcon TRC-50I IX fundus camera equipped with the digital Image Net image system, version 2.14 (Topcon, Tokyo, Japan). An informed written consent was signed by all participants and the study design was approved by the Bioethics Committee of the Medical University of Warsaw. A structured questionnaire was used to determine demographic characteristics, family history of AMD (first-degree relatives), living environment (rural or urban areas) and life-style, including smoking and alcohol use. Each subject donated a venous blood sample of ~5 ml, 250 μl of which was used for genomic DNA extraction. Blood samples of all patients and controls were collected into ethylenediaminetetraacetic acid (EDTA) tubes and stored at −20°C until further use. All samples were coded after collection of blood and completing the questionnaire. Data on disease status, sex, age, smoking history, living environmental, and family history of AMD of the subjects enrolled in this study are presented in Table 1.

**Table 1:** Characteristics of AMD patients and AMD-free control subjects enrolled in the study

Characteristics	Controls		AMD		*p*
	Number	Frequency	Number	Frequency	
Disease status	116				
No AMD					
Dry AMD			100	0.37	
Wet AMD			170	0.63	
Sex					
Females	82	0.78	179	0.66	**0.0316**
Males	23	0.22	92	0.34	
Age					
≤60	31	0.30	32	0.12	**<0.001**
≥61	74	0.70	239	0.88	
Mean ± SD	68.20 ± 10.95		72.46 ± 8.51		
Range	50–88		52–93		
Smoking					
Ever	32	0.41	68	0.41	0.9251
Never	46	0.59	99	0.59	
Living environment					
Rural	32	0.38	50	0.30	0.2880
Urban	52	0.62	114	0.70	
AMD in family					
Yes	3	0.04	33	0.20	**<0.001**
No	81	0.96	130	0.80	

*p* values for a two-sides χ^2^ test, *p* values <0.05 are in *bold*

### SNP selection and primers design

There are many SNPs in the *IRP1* and *IRP2* genes listed in the dbSNP short genetic variations database in the NCBI http://www.ncbi.nlm.nih.gov/snp, but none of them has annotated any clinical significance and were not able to find any evidence for this. Our selection of polymorphisms was made on the basis of their location in the 5′ regulatory region, which usually contains the promoter. The phenotypic consequences of such DNA sequence changes may be associated with the difference in the transcription level, which can be quantitatively assessed in future research. Primers were designed using the *IRP1* and *IRP2* genomic sequence found at http://www.ensembl.org/Homo_sapiens/Gene/Sequence?g=ENSG00000122729;r=9:32384601-32450834 and http://www.ensembl.org/Homo_sapiens/Gene/Summary?g=ENSG00000136381;r=15:78730531-78793795;t=ENST00000258886 and Primer3Plus software (http://www.bioinformatics.nl/cgi-bin/primer3plus/primer3plus.cgi) for *IRP2* SNP and Web-based allele-specific primer software (http://bioinfo.biotec.or.th/WASP/index/wasp ) for *IRP1* SNP.

### DNA preparation and storage

Genomic DNA was obtained from a 250 μl aliquot of blood using a commercially available AxyPrep^™^ Blood genomic DNA Miniprep Kit (Axygen Biosciences, Union City, CA, USA), according to the manufacturer’s instructions. Genomic DNA was directly isolated from the white blood cells. DNA purity and concentration were determined spectrophotometrically at 260 and 280 nm. Each DNA sample was stored in TE buffer (5 mM Tris–HCl, 0.1 mM EDTA, pH 8.5) at −20°C until analysis.

### Genotyping

**Fig. 1:**
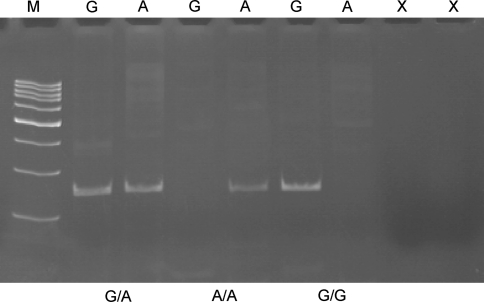
Genotypes of the g.32373708 G>A-*IRP1* polymorphism (rs867469) determined by the allele-specific oligonucleotide-PCR (ASO-PCR) method and analyzed by a 8% polyacrylamide gel electrophoresis stained with ethidium bromide and viewed under UV light. *Lane M* displays GeneRuler ^™^ 100 bp molecular weight marker, *lanes designated G or A* show the results of amplification with primer specific to either the G or A allele, respectively and *lane X* shows a negative control comprising reaction mixture without target DNA. *Genotypes* are indicated in the lower part of the picture

The genotypes of the g.32373708 G>A-*IRP1* polymorphism were determined by the allele-specific oligonucleotide-PCR method. PCR was performed in a 25 μl reaction volume. The reaction mixture contained 50 ng of genomic DNA, 1 U Biotools DNA polymerase (Biotools, Madrid, Spain), 1 × reaction buffer [750 mM Tris–HCl, pH 9.0, 500 mM KCl, 200 mM (NH4)_2_SO_4_], 0.2 mM of each dNTP, 1.5 mM MgCl_2_, 0.25 μM of each primers (Metabion, Martinsried, Germany). The primers designed to detect the g.32373708 G>A SNP were as follows: allele-specific sense oligonucleotides 5′-TGCACACCTGCAAAGAAG-3′ for G variant and 5′-TGCACACCTGCAAAGAAA-3′ for A variant and antisense oligonucleotide 5′-CTAGATGAAAGGTGGTGAGG-3′. The allele-specific oligonucleotide-restriction fragment length polymorphism (ASO-PCR) conditions were as follows: 5 min of initial denaturation at 95°C, followed by 30 cycles of 30 s denaturation at 95°C, 30 s annealing at 56°C and 1 min extension at 72°C. The final extension step at 72°C for 5 min was also included. The PCR products (237 bp) were fractionated by electrophoresis on a 8% polyacrylamide gel, stained with ethidium bromide and viewed under UV light. Fig. 1 presents a representative gel from analysis of this polymorphism.

**Fig. 2:**
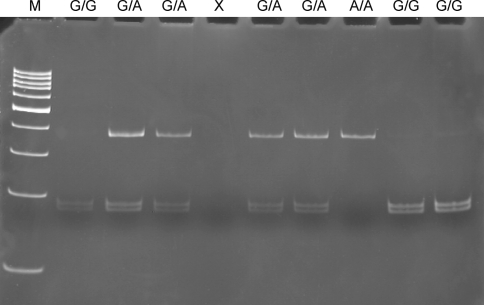
Genotypes of the g.49520870 G>A-*IRP2* polymorphism (rs17483548) determined by the PCR-RFLP detection and analyzed by a 8% polyacrylamide gel electrophoresis stained with ethidium bromide and viewed under UV light. *Lane M* shows GeneRuler ^™^ 100 bp molecular weight marker, *lane X* shows a negative control comprising reaction mixture without target DNA, all remaining lanes present genotypes indicated in the upper part of the picture

The polymerase chain reaction-restriction fragment length polymorphism (PCR-RFLP) method was used to determine the genotypes of the g.49520870 G>A-*IRP2* polymorphism. PCR assay was performed in a total reaction volume of 25 μl containing the same chemicals as in the previous analysis except for primers. 360 bp length fragments containing polymorphic site were amplified using the following primers: sense 5′-CCCCCACTTGAAAACACG-3′ and antisense 5′-AGATCGTCGGACAGGAAAAC-3′. The PCR profile contained an initial denaturation step for 5 min at 95°C, 30 cycles at 95°C for 30 s, 30 s at 60°C annealing temperature and 60 s at 72°C and the final extension step for 5 min at 72°C. After amplification, the 360 bp PCR products were analyzed on a 3% agarose gel and digested with 3 U of *Bsp*TI (*Afl*II) restriction endonuclease (Fermentas, Hanover, MD, USA) in a final volume of 15 μl for 16 h at 37°C. PCR products with a G at the polymorphic site were digested into two 183 and 177 bp fragments, while those with A were not because of the absence of a *Bsp*TI (*Afl*II) restriction site. The G/G genotype produced two fragments (183, 177 bp), whereas the G/A genotype produced three fragments (360, 183, and 177 bp) and the homozygote A/A resulted in only one fragment 360 bp. Digested PCR products were separated by electrophoresis on a 8% polyacrylamide gel and visualized by ethidium bromide staining using a GeneRuler ^™^ 100 bp (Fermentas, Hanover, MD, USA) as a size marker. A representative gel for this polymorphism is presented in Fig. 2. All PCR amplifications were conducted in a C1000 Thermal Cycler (Bio-Rad Laboratories, Hercules, CA, USA). Positive and negative (no template) controls were included in all sets. For quality control, we randomly selected 10% samples for each of the two SNPs to perform repeat assays and the results were 100% concordant.

### Statistical analysis

All statistical analyzes were performed using STATISTICA 9.0 software (Statsoft, Tulsa, OK, USA) and SigmaPlot v11.0 software (Systat Software, Inc., San Jose, CA, USA). To compare the distributions of demographic variables and selected risk factors between patients and controls Chi-square test was used. Hardy–Weinberg equilibrium (HWE) was checked using Chi-square test to compare the observed genotype frequencies with the expected frequencies among the case and control subjects. The χ^2^ analysis was also used to test the significance of the differences between distributions of genotypes and alleles in AMD patients and controls. The association between case–control status and each polymorphism, measured by the odds ratio (OR) and its corresponding 95% confidence interval (CI), was estimated using an unconditional multiple logistic regression model, both with and without adjustment for sex, age, smoking habit, living environment (rural versus urban areas), and family status of AMD. Smoking habit was categorized in terms of never smokers and smokers (including current and former). Stratified analysis according to age, sex, and family status of AMD was also conducted. To study a possible gene-environment interaction, the patients and controls were divided into subgroups depending on sex, family history and age. Unconditional logistic regression analyzes were also performed to assess the association between genotypes and risk for AMD after stratification of the individuals according to sex, age, and family status of AMD.

## Results

### Characteristics of the study population and relationship between demographic, environmental, life-style factors, and family history and the risk of AMD independent of genotype

The characteristics of the patients with AMD and AMD-free controls involved in this study are presented in Table 1. The mean ± SD age was 72.46 ± 8.51 years for the patients (range 52–93) and 68.29 ± 10.95 years for the controls (range 50–88) and 34% of the patients and 23% of the controls were men, whereas 66% of the patients and 82% of the controls were women. There were significantly more subjects with negative family history for AMD among the controls than patients (96 vs. 80%, *p* *<* 0.001). Therefore, these variables were further adjusted in the multivariate logistic regression model to control for possible confounding factors of the main effects of the polymorphisms. Moreover, we explored the relationships between age, sex, smoking, living environment and family history of AMD and the risk of AMD independently of genotype. We compared AMD patients and controls according to these parameters (Table 2). Our results suggest that male sex (OR 1.83, 95% CI 1.08–3.10; *p* = 0.024), age (7.42, 95% CI 2.62–21.02, *p* *<* 0.001) and first-degree relatives with AMD (OR 6.85, 95% CI 2.04–23.08; *p* = 0.002) significantly increased the risk of the disease.

**Table 2:** Risk of AMD associated with age, sex, smoking, living environment and family history of AMD

Characteristics	Controls	AMD	OR (95% CI)	*p*
	Number (frequency)	Number (frequency)		
Age				
From point			**1.05** (**1.02–1.07**)	**<0.001**
From range			**7.42** (**2.62–21.02**)	**<0.001**
Sex				
Females	82 (0.78)	179 (0.66)	**0.55** (**0.32–0.92**)	**0.024**
Males	23 (0.22)	92 (0.34)	**1.83** (**1.08–3.10**)	**0.024**
Smoking				
Ever (current, former)	32 (0.41)	68 (0.41)	0.99 (0.57–1.71)	0.964
Never	46 (0.59)	99 (0.59)	0.91 (0.53–1.58)	0.748
Living environment				
Rural	32 (0.38)	50 (0.30)	0.71 (0.41–1.24)	0.229
Urban	51 (0.62)	114 (0.70)	1.40 (0.81–2.44)	0.229
AMD in family				
Yes	3 (0.04)	33 (0.20)	**6.85** (**2.04–23.08**)	**0.002**
No	81 (0.96)	130 (0.80)	**0.15** (**0.05–0.51**)	**0.002**

*p* values <0.05 along with corresponding ORs are in *bold*

*OR* odds ratio, 95% CI

### Polymorphisms of the *IRP1* and *IRP2* genes, gene–gene interaction and AMD occurrence and progression

The genotype and allele distributions of the g.32373708 G>A-*IRP1* and g.49520870 G>A-*IRP2* polymorphisms in AMD patients and controls are summarized in Table 3. The observed genotype frequencies of the g.49520870 G>A-*IRP2* SNP were all in agreement with the HWE calculated for the cases and controls (*p* > 0.05, data not shown). As shown in Table 3 the difference in the frequency distributions of genotypes of the g.32373708 G>A-*IRP1* SNP between the cases (AMD, dry and wet forms of the disease) and controls was statistically significant (*p* *<* 0.05). An association between AMD, dry and wet forms of AMD and the G/G genotype of the g.32373708 G>A-*IRP1* polymorphism was found (adjusted OR 3.40, 4.15, and 2.75, respectively). On the other hand, the G/A genotype reduced the risk of AMD as well as dry form of AMD or wet form of AMD, separately (adjusted OR 0.23, 0.21, and 0.26, respectively). Furthermore, in the dry form of AMD, the G allele of the g.49520870 G>A-*IRP2* SNP increased the risk of the disease (OR 1.51) and the A/A genotype and the A allele decreased such risk (OR 0.43 and 0.66).

**Table 3:** Distribution of genotypes, frequency of alleles of the g.32373708 G>A-*IRP1* and g.49520870 G>A-*IRP2* polymorphisms and odds ratio (OR) with 95% CI (95% CI) in patients with AMD, the disease in its dry and wet forms and individuals without visual disturbances (controls)

Genotype/allele	Controls	AMD	Crude OR (95% CI)	Adjusted OR^a^ (95% CI)	Dry AMD	Crude OR (95% CI)	Adjusted OR^a^ (95% CI)	Wet AMD (95% CI)	Crude OR (95% CI)	Adjusted OR^a^ (95% CI)
	Number (frequency)	Number (frequency)			Number (frequency)			Number (frequency)		
g.32373708 G>A, rs867469	*n* = 98	*n* = 269			*n* = 99			*n* = 170		
G/G	22 (0.23)	101 (0.38)	**2.07** (**1.21–3.57**)	**3.40** (**1.71–6.78**)	35 (0.35)	**1.89** (**1.01–1.69**)	**4.15** (**1.81–9.51**)	66 (0.38)	**2.19** (**1.24–3.86**)	**2.75** (**1.31–5.76**)
G/A	67 (0.68)	95 (0.35)	**0.25** (**0.15–0.41**)	**0.23** (**0.23–0.43**)	36 (0.36)	**0.26** (**0.14–0.48**)	**0.21** (**0.09–0.45**)	59 (0.35)	**0.25** (**0.14–0.42**)	**0.26** (**0.13–0.52**)
A/A	9 (0.09)	73 (0.27)	3.68 (1.76–7.69)	2.25 (0.96–5.28)	28 (0.28)	3.90 (1.73-8.80)	1.97 (0.72–5.39)	45 (0.27)	3.56 (1.66-7.65)	2.43 (0.97-6.13)
		χ^2^ = 33.024, *p* *<* **0.0001**			χ^2^ = 22.047, *p* *<* **0.0001**			χ^2^ = 29.278, *p* *<* **0.0001**		
G	172 (0.82)	297 (0.55)	0.94 (0.68–1.31)	1.35 (0.89–2.05)	106 (0.53)	0.88 (0.59–1.31)	0.16 (0.93–2.85)	191 (0.56)	0.98 (0.69–1.40)	1.21 (0.75–1.97)
A	38 (0.18)	241 (0.45)	1.06 (0.76–1.47)	0.74 (0.49–1.12)	92 (0.47)	1.13 (0.76–1.69)	0.62 (0.35–1.08)	149 (0.44)	1.01 (0.71–1.45)	0.82 (0.51–1.33)
g.49520870 G>A, rs17483548	*n* = 116	*n* = 263			*n* = 100			*n* = 163		
G/G	40 (0.34)	107 (0.41)	1.30 (0.83–2.05)	0.93 (0.52–1.65)	44 (0.44)	1.50 (0.86–2.60)	1.24 (0.61–2.53)	63 (0.38)	1.20 (0.73–1.97)	0.81 (0.42–1.58)
G/A	52 (0.45)	121 (0.46)	1.05 (0.68–1.63)	1.16 (0.66–2.02)	46 (0.46)	1.05 (0.61–1.80)	1.05 (0.52–2.11)	75 (0.46)	1.05 (0.65–1.70)	1.21 (0.65–2.28)
A/A	24 (0.13)	35 (0.13)	0.59 (0.33–1.04)	0.87 (0.42–1.84)	10 (0.10)	**0.43** (**0.19–0.94**)	0.59 (0.21–1.65)	25 (0.15)	0.69 (0.37–1.29)	0.99 (0.43–2.26)
		χ^2^ = 3.640, *p* = 0.1620			χ^2^ = 5.166, *p* = 0.0756			χ^2^ = 1.445, *p* = 0.4855		
G	132 (0.57)	335 (0.64)	1.33 (0.97–1.81)	1.00 (0.67–1.56)	134 (0.67)	**1.51** (**1.02–2.24**)	1.27 (0.77–2.11)	201 (0.62)	1.22 (0.87–1.70)	0.91 (0.58–1.43)
A	100 (0.43)	191 (0.36)	0.76 (0.56–1.04)	0.99 (0.67–1.50)	66 (0.33)	**0.66** (**0.44–0.97**)	0.78 (0.47–1.30)	125 (0.38)	0.83 (0.59–1.15)	1.10 (0.70–1.71)

*p* values for a two-sides χ^2^ test, significant *p* and ORs are in *bold*

^a^OR adjusted for age, sex, AMD in family

–, not estimated

We also evaluated the associations between the occurrence of AMD and combined genotypes of the g.32373708 G>A-*IRP1* and g.49520870 G>A-*IRP2* polymorphisms. The distribution of combined genotypes of these polymorphisms is shown in Table 4.

**Table 4:** Distribution of combined genotypes of the g.32373708 G>A-*IRP1* and g.49520870 G>A-*IRP2* polymorphisms and odds ratio (OR) with 95% confidence interval (95% CI) in patients with AMD as well as its dry and wet forms and individuals without visual disturbances (controls)

Genotype	Controls (*n* = 79)	AMD (*n* = 254)	Crude OR (95% CI)	Adjusted OR^a^ (95% CI)	Dry AMD (*n* = 99)	Crude OR (95% CI)	Adjusted OR^a^ (95% CI)	Wet AMD (*n* = 155)	Crude OR (95% CI)	Adjusted OR^a^ (95% CI)
	Number (frequency)	Number (frequency)			Number (frequency)			Number (frequency)		
G/G–G/G	6 (0.07)	38 (0.15)	2.14 (0.87–5.27)	2.94 (0.91–9.55)	17 (0.17)	2.52 (0.94–6.74)	4.46 (1.21–16.41)	21 (0.13)	1.90 (0.74–4.91)	2.03 (0.55–7.56)
G/G–G/A	5 (0.06)	45 (0.18)	**3.19** (**1.22–8.34**)	**3.52** (**1.20–10.30**)	14 (0.14)	2.44 (0.84–7.09)	3.22 (0.91–11.40)	31 (0.20)	**3.70** (**1.38–9.93**)	3.05 (1.01–9.37)
G/G–A/A	7 (0.09)	14 (0.05)	0.60 (0.23–1.54)	1.05 (0.30–3.67)	4 (0.04)	0.43 (0.12–1.54)	1.14 (0.26–5.08)	10 (0.06)	0.71 (0.26–1.94)	1.01 (0.25–4.07)
G/A–G/G	25 (0.32)	36 (0.14)	**0.36** (**0.21–0.64**)	**0.23** (**0.10–0.51**)	15 (0.15)	**0.39** (**0.19–0.80**)	**0.26** (**0.09–0.68**)	21 (0.13)	**0.34** (**0.17–0.65**)	0.25 (0.11–0.61)
G/A–G/A	23 (0.30)	40 (0.16)	**0.46** (**0.25–0.82**)	0.48 (0.22–1.04)	17 (0.17)	0.50 (0.25–1.03)	0.51 (0.19–1.36)	23 (0.15)	**0.42** (**0.22–0.82**)	0.51 (0.21–23)
G/A–A/A	7 (0.09)	12 (0.05)	0.51 (0.20–1.34)	1.25 (0.37–4.15)	4 (0.04)	0.43 (0.12–1.53)	0.75 (0.15–3.82)	8 (0.05)	0.56 (0.19–1.60)	1.55 (0.42–5.75)
A/A–G/G	3 (0.04)	31 (0.12)	3.52 (1.05–11.85)	2.38 (0.51–11.16)	12 (0.12)	3.49 (0.95–12.84)	2.48 (0.46–13.24)	19 (0.12)	**3.54** (**1.01–12.35**)	2.40 (0.47–12.40)
A/A–G/A	2 (0.02)	31 (0.12)	5.35 (1.25–22.90)	3.78 (0.81–17.60)	14 (0.14)	**6.34** (**1.39–28.80**)	3.12 (0.55–17.55)	17 (0.11)	**4.74** (**1.06–21.07**)	4.30 (0.86–21.06)
A/A–A/A	1 (0.01)	7 (0.03)	2.21 (0.27–18.25)	0.23 (0.01–6.41)	2 (0.02)	1.61 (0.14–18.01)	–	5 (0.03)	2.60 (0.30–22.64)	0.45 (0.02–11.61)

Significant ORs are in *bold*; –, not estimated

^a^OR adjusted for age, sex, AMD in family

The presence of the A/A–G/A genotype of both polymorphisms increased the risk of AMD as well as its dry or wet form, separately (OR 5.35, 6.34, and 4.74, respectively), whereas the presence of the G/A–G/G genotype decreased such risk (OR 0.36, 0.39, and 0.34, respectively).

Furthermore, the G/A–G/A genotype may have a protective effect against wet AMD and AMD (OR 0.46 and 0.42, respectively). In the wet form of AMD and AMD, the G/G–G/A and A/A–G/G genotypes increased the risk of the disease (OR 3.70, 3.54, 3.19, and 3.52, respectively).

Additionally, we examined the distribution of genotypes/combined genotypes and alleles of the g.32373708 G>A-*IRP1* and g.49520870 G>A-*IRP2* polymorphisms in the group of wet AMD patients in comparison with dry AMD patients (data not shown). Such comparison was considered as a measure of AMD progression. We did not find any association between the progression of AMD and genotypes/combined genotypes and alleles of both polymorphisms.

### Stratification and interaction analysis of *IRP1* and *IRP2* genotypes and the risk of AMD

The association between AMD and the tested SNPs was subjected to stratification analysis, but only data for the g.32373708 G>A-*IRP1* are presented in Table 5 because such analysis for other polymorphism did not yield any significant result. In stratification analysis, the G/G genotype was associated with a significantly increased risk of AMD in subgroups of subjects of 61 years and older (adjusted OR 2.44) and women (adjusted OR 3.54). On the other hand, the G/A genotype reduced the risk of AMD in subgroups of subjects older than 61 years (adjusted OR 0.26), women (adjusted OR 0.20) and smokers (adjusted OR 0.19). Moreover, the occurrence of AMD was correlated with the presence of the A/A genotype in women (adjusted OR 2.87).

**Table 5:** Distribution of genotypes of the g.32373708 G>A-*IRP1* polymorphism stratified by age, sex and smokers in patients with dry AMD as compared with individuals without visual disturbances (controls)

Genotype/allele	Controls	AMD	Adjusted OR^a^	*p*
	Number (frequency)	Number (frequency)		
Age >61	*n* = 67	*n* = 235		
G/G	15 (0.22)	88 (0.37)	**2.44** (**1.13–5.25**)	**0.022**
G/A	46 (0.69)	86 (0.37)	**0.26** (**0.13–0.53**)	**<0.001**
A/A	6 (0.09)	61 (0.26)	**2.87** (**1.03–7.95**)	**0.043**
G	76 (0.57)	262 (0.56)	1.09 (0.69–1.73)	0.699
A	58 (0.43)	208 (0.44)	0.91 (0.57–1.44)	0.699
Women	*n* = 77	*n* = 173		
G/G	19 (0.25)	68 (0.39)	**3.54** (**1.60–7.84**)	**0.002**
G/A	52 (0.67)	60 (0.35)	**0.20** (**0.11–0.42**)	**<0.001**
A/A	6 (0.08)	45 (0.26)	2.76 (0.95–7.98)	0.060
G	90 (0.58)	196 (0.57)	1.36 (0.82–2.24)	0.227
A	64 (0.42)	150 (0.43)	0.73 (0.44–1.21)	0.227
Smokers	*n* = 31	*n* = 68		
G/G	2 (0.06)	24 (0.36)	2.58 (0.77–8.59)	0.122
G/A	24 (0.77)	22 (0.32)	**0.19** (**0.06–0.58**)	**0.003**
A/A	5 (0.16)	22 (0.32)	4.73 (0.94–23.69)	0.059
G	34 (0.55)	70 (0.48)	0.94 (0.46–1.92)	0.883
A	28 (0.45)	66 (0.52)	1.05 (0.51–2.14)	0.883

*p* values <0.05 along with corresponding ORs are in *bold*

^a^OR adjusted for age, sex and smoking

## Discussion

We showed that age, sex, and family status of AMD were significant risk factors in the development of AMD, which is in agreement with previous results obtained by others [30, 31]. However, we did not find any association between smoking and AMD. At present we have not any direct explanation of this fact except that smoking may be one of the factor of AMD pathogenesis and not the sole reason of AMD and the contribution of this factor depends on the population [32]. Smoking status in our questionnaire was defined as a smoker or non-smoker without taking into account passive smoking. It was shown that passive smoking is associated with an almost twofold increase in the risk of AMD for non-smokers having lived with smokers for 5 years or more. This might explain, at least in part, our apparently controversial data on association between AMD and smoking [33]. Therefore, more detailed characteristics of the population enrolled in the present study might have shed on this apparent lack of agreement with the established point of view.

Although the pathogenesis of AMD is not completely understood, a growing body of evidence suggests that oxidative stress and free radical damage may mediate or exacerbate macular degeneration [34]. Oxidative damage is implicated in several retinal diseases, including retinal degeneration and AMD [35–37]. In a large clinical trial, patients with dry AMD given dietary supplements of antioxidants and zinc had reduced progression to advanced AMD, suggesting that oxidative stress is somehow involved in its pathogenesis [38]. Iron is suggested as a potential source of oxidative radicals associated with degenerative processes affecting the central nervous system and the retina [34, 39, 40]. Hahn et al. [41] found that AMD-affected maculas had significantly increased total iron concentration compared with age-matched controls, suggesting that iron accumulation might play a role in this disease. Although iron can cause oxidative tissue damage through the Haber–Weiss and Fenton reactions, it is also an essential for several metabolic pathway. Homeostatic regulation of ferrous iron levels is critical for meeting physiologic demand while preventing the toxicity associated with iron overload [34, 42]. Several studies have confirmed that iron overload may play a crucial role in the pathogenesis of AMD [15, 34]. Balanced iron homeostasis is coordinated largely at the posttranscriptional level via the interaction of either of two IRPs with *cis*-regulatory RNA motifs—IREs located in the 5′ or 3′ untranslated regions (UTR) of mRNAs encoding proteins of iron uptake [transferrin receptor 1 (TfR1) and divalent metal transporter-1 (DMT-1)], export (ferroportin), utilization (5-aminolevulinate synthase) or storage (ferritin H- and L-chains) [43]. In mammals, two homologous IRPs have been identified. *IRP1* also known as the cytosolic aconitase (ACO1) has two mutually exclusive functions. When iron is abundant, *IRP1* assembles an Fe–S cluster and has aconitase activity, whereas—when iron is scarce, the apoprotein without its Fe–S cluster acquires IRE-binding activity [44]. *IRP2* also called IREB2 (iron-responsive element binding protein 2) shares 79% homology with *IRP1* but lacks aconitase activity [45]. Mice with combined total and constitutive deficiency of both IRPs show embryonic lethality, suggesting that the IRPs are fundamental for life [46–48]. In both iron deficiency and excess, IRP-mediated regulation rapidly restores the physiological cytosolic iron level. Under low-iron conditions, IRPs binds to IREs present in the 5′ UTR of mRNAs, such as in ferritin heavy and light chains and inhibits translation and concurrently IRPs bind to mRNAs containing IREs in the 3′ UTR, such as TfR1 and DMT1, resulting in increasing RNA’s stability and levels. And vice versa, under iron high conditions, IRP binding activity to IREs is reduced. Thus, under iron-poor condition, IRPs upregulate genes to increase iron uptake and under iron rich condition, IREs upregulate genes that are required for iron storage [49–51]. The importance of normal iron homeostasis is underlined by the fact that retinal dysfunction has been observed in some pathological conditions due to the lack of iron or an excess of iron [52–54].

Some results demonstrate that multiple genes and proteins are involved in development of AMD. AMD can be genetically associated with multiple susceptibility loci, i.a. 1q32 (complement factor H, CFH), 10q26 (ARMS2), 6p21.3 (complement factor B, *BF*; complement component 2, C2), 19p13.3–13.2 (complement component 3, C3) [55–61]. As mentioned, both genetic predispositions and environmental factors, such as smoking and ultraviolet rays, play important roles in pathogenesis of AMD. Interestingly, hereditary diseases of iron overload are caused by mutations in genes of both regulation and usage [62, 63]. Furthermore, there is a growing body of evidence that SNPs associated with inflammation, oxidative stress, angiogenesis and other pathological processes have been linked to AMD [61]. Because iron overload has been implicated in AMD, IRPs are a strong potential candidate for polymorphisms that could play a role in AMD. We assessed whether the g.32373708 G>A polymorphism in the *IRP1* gene and the g.49520870 G>A polymorphism in the *IRP2* gene increase the risk of AMD. To our knowledge, the g.32373708 G>A-*IRP1* polymorphism and the g.49520870 G>A-*IRP2* polymorphism have not been studied in AMD patients so far. We observed, for the first time, that the occurrence of AMD was positively correlated with the presence of the G/G genotype of the *IRP1* SNP and of the G allele of the *IRP2* SNP.

In conclusion, our work shows that genetic polymorphisms of the *IRPs* genes may be associated with development of AMD. These findings may be helpful in increasing our understanding of the etiology of AMD. Identification of genetic risk factors for AMD is the first step towards earlier detection and prevention, and in the future, better treatments.
